# Travel behaviour change in old age: the role of critical incidents in public transport

**DOI:** 10.1007/s10433-015-0358-8

**Published:** 2015-12-15

**Authors:** Catherine Sundling, Mats E. Nilsson, Sara Hellqvist, Leslie R. Pendrill, Ragne Emardson, Birgitta Berglund

**Affiliations:** 1 Gösta Ekman Laboratory, Department of Psychology, Stockholm University, 106 91 Stockholm, Sweden; 2 Department of Psychology, Stockholm University, 106 91 Stockholm, Sweden; 3 SP–Technical Research Institute of Sweden, P.O. Box 857, 501 15 Borås, Sweden; 4 Institute of Environmental Medicine, Karolinska Institutet, P.O. Box 210, 171 77 Stockholm, Sweden

**Keywords:** Older people, Travel behaviour, Public transport, Critical incidents, Qualitative research

## Abstract

Older people’s travel behaviour is affected by negative or positive critical incidents in the public transport environment. With the objective of identifying such incidents during whole trips and examining how travel behaviour had changed, we have conducted in-depth interviews with 30 participants aged 65–91 years in the County of Stockholm, Sweden. Out of 469 incidents identified, 77 were reported to have resulted in travel behaviour change, 67 of them in a negative way. Most critical incidents were encountered in the physical environment on-board vehicles and at stations/stops as well as in pricing/ticketing. The findings show that more personal assistance, better driving behaviour, and swift maintenance of elevators and escalators are key facilitators that would improve predictability in travelling and enhance vulnerable older travellers’ feeling of security. The results demonstrate the benefit of involving different groups of end users in future planning and design, such that transport systems would meet the various needs of its end users.

## Introduction

Because of the ageing populations in many European countries, the proportion of journeys made by older people is expected to increase (Myck 2015). For example, by 2060, almost 25 % of the Swedish population is expected to be more than 65 years old, compared with 19 % in 2011 (Statistics Sweden 2012). Living longer and maintaining an active lifestyle longer create possibilities and wishes for a variety of activities (Hjorthol 2013). But, with increasing age, functional limitations become more common, and many older adults will have acquired more than one such limitation (Sundling et al. 2014a), which may complicate travelling.

Webber et al. (2010) define mobility as the ability to move oneself within environments that extend beyond one’s home to the neighbourhood and other regions beyond. Mobility is determined by cognitive, psychosocial, physical, environmental, and financial influences. It may reduce the risk of social exclusion, which in turn enhances well-being (Stanley et al. 2011). Older adults constitute a heterogeneous group with regard to mobility: Those aged 75+ are less satisfied with their mobility opportunities than those aged 65–74 years (Mollenkopf et al. 2011). Siren and Hakamies-Blomqvist (2006) show that people without a driver’s licence, those living in rural areas, and women, experience unfulfilled travel needs. Being less likely to possess a driver’s licence or a car than men, women may be more dependent on an accessible public transport. Even if, in some countries, the life-expectancy gap between men and women is narrowing, the majority of the older old are, and will be, women (Hjorthol 2013; Shergold et al. 2015).

Travel frequency decreases with age, especially from 75 (Heikkinen and Henriksson 2013). Boschmann and Brady (2013) found that, with increasing age (above 60+), travellers make fewer and shorter trips; women make fewer and shorter trips than men, and persons with disabilities make the fewest trips as compared to all other persons. Moreover, the distance travelled increases with household size. Driving cessation is associated with negative consequences such as increased dependency, social isolation, depression, and increased mortality risk (Webber et al. 2010). In a sample of questionnaire respondents aged 65–85 years, Sundling et al. (2014a) found that 41 % wished they could travel by train more often and those with high functional ability travelled more often than those with low. Schmöcker et al. (2008) show that public transport travelling for shopping purposes is adversely associated with functional limitations.

Various public transport barriers or facilitators have been identified for older adults/or persons with functional limitations. Examples are ticket prices (Su et al. 2009), boarding/alighting, distance to bus stop, and (in)security if travelling alone (Wretstrand et al. 2009). Iwarsson and Ståhl (1999) showed that the possibility to participate in society is perceived to be reduced due to barriers encountered on the way to and from the bus stop, or while entering or alighting the bus, by as much as 75 % in a group of older or Special Transport Service^1^-entitled respondents. Bus stop (but not rail and underground station) density will increase older adults’ travel frequency with the same travel mode (Schmöcker et al. 2008). Moreover, short walking distances *within* stations and reliability of service have been identified as facilitators. For travellers with cognitive deficits, serial tasks and high complexity of the travel environment may be demanding (Rosenkvist et al. 2009). For this group, compared to cognitively healthy persons, complex out-of-home activities are associated with higher negative affect (Wettstein et al. 2014).

Accessibility is dependent on the physical environment and on person factors such as functional limitations (Jensen et al. 2002). In the ecological model by Lawton and Nahemow’s and further developed by Wahl et al. (2012), a balance between environmental pressure and a person’s ability may be reached if one, or both, are changed (Jensen et al. 2002). The present paper builds on a reciprocal model of accessibility involving a person’s functional ability (functional limitations inclusive), his/her travel behaviour, and the barriers/facilitators encountered (Sundling et al. 2014b). Therefore, in order to understand the *concept of accessibility*, knowledge on the characteristics of both the person and the environment are necessary.

It is not evident, ab initio, how barriers and/or facilitators encountered link to future travel behaviour. We have selected a qualitative research approach since it might provide a deeper understanding of people’s experiences compared to a purely quantitative approach. Qualitative research builds on the travellers’ actual experiences and may help to pinpoint important occurrences during trips, as experienced by the travellers themselves. Qualitative research may therefore facilitate insights into their decision processes (Edvardsson 1998). Barriers/facilitators in travelling might be perceived differently by different travellers. Therefore, a focus on the individual might help to reveal needs that would not be discerned in the population at large. Notably, there is a lack of knowledge on how persons with functional limitations, in general, perceive public transport travelling; especially in an “entire journey perspective” (The Swedish Parliament 2014).

The present research addresses public transport, including rail-bound modes and buses, but primarily railway travelling. All public travel modes have been included, provided that at least part of the trip was by rail. In-depth interviews were conducted using the Critical Incident Technique (CIT). This technique is used to describe an activity that has actually happened (Butterfield et al. 2005) rather than attitudes and has previously been used with older adults (Marcinowicz et al. 2014). Because open questions are posed, a broad spectrum of experiences may be captured without pushing the participants’ thoughts in a certain direction (Flanagan 1954; Edvardsson 1998). The CIT may also identify rare and particularly decisive events in the public transport system that otherwise might have been missed. The aims of the study were to identify *critical incidents* in public transport travelling and to examine how these critical incidents might affect *travel behaviour*.

## Method

### Participants

Participants were recruited from the County of Stockholm. All were experienced public transport travellers. A heterogeneous sample was chosen purposely to provide a diversity of perceived difficulties in travelling. The participants varied in age, gender, *kind* of functional limitation, *degree* of functional ability, travel frequency, travel modes used, area of residency (city/suburb), household size, and car possession. The first author selected the participants. Ten were recruited from a previous railway accessibility study (Sundling et al. 2014a) and twenty by advertising in a local inner-city newspaper, through the municipality’s care for the elderly (inner city) and by snowball sampling, i.e. participants recruited through referrals among persons with characteristics of interest for the research (Biernacki and Waldorf 1981). The participants recruited by snowball sampling and from previous research, were living in different parts of the larger metropolitan area.

Inclusion criteria were age (65 or older), travel behaviour (last 2 years active railway travelling, all modes) and “transportation disability” [functional limitation (15 kinds) *or* reduced functional ability (on a 5-category scale), *or* railway travelling with luggage/children under 6 years]. A questionnaire on the inclusion criteria was constructed and used to select participants for individual in-depth interviews.

### Critical incidents

The Critical Incident Technique (CIT; see Flanagan 1954) is explorative. CIT may provide basic knowledge for theory development. It has often been used for appraising system performance from the consumer’s perspective (Kolbe and Burnett 1991). The CIT procedure was developed for collecting events and human behaviours and to categorise them to make them useful for addressing practical problems (Bitner et al. 1990).

A *critical incident* is an event that is perceived as particularly satisfying or dissatisfying. In this research, an incident is referred to as *critical* if it has had a “high influence” on travel behaviour. Our inclusion criteria for an incident were: an occurrence encountered by the interviewee, affecting the experience of a public transport journey, *at least in part* made by railway during the last 2 years in Sweden (including all rail-bound modes; long-distance trains, commuter trains, underground, and local trains & trams). This time limit was set to ensure that transport-system changes had been minor, and that the incidents could be more easily remembered than if they had happened several years ago. The whole trip is included, from planning to destination. Each single event is viewed as a separate incident and analysed separately. A previous incident may act as an *antecedent,* potentially influencing the perception of a subsequent incident. In this research, antecedents are viewed as long-term and short-term occurrences or expectations preceding the incident. Long-term travel behaviour is an outcome, at least in part determined by the traveller’s perception of the incident encountered.

### Data collection

The first author served as interviewer. The interviewees were informed about research goals and anonymity. With informed consent, they were then asked to report all *incidents* they could remember. Incidents per interview varied in number from 1 to 47 and interview duration from 15 to 150 min. Descriptive validity was ensured by written answers and recordings (cf. Butterfield et al. 2005; Maxwell 1992). Occasional and repeated incidents were collected. An interviewee would give a detailed description of each incident. Open questions were about antecedents, course and reactions (cognitive, emotional and/or behavioural) to the incident; and, in the case of negative incidents, how to reduce them (see e.g. Butterfield et al. 2005). Each incident was classified as *positive or negative* and scaled on a 3-point category scale regarding its *influence* on participant travel behaviour. The response categories were: “1: no influence”, “2: low influence” or “3: high influence”. A “high influence” meant that travel behaviour had actually changed because of the incident. After 15 completed interviews, no new incident categories emerged, which indicated saturation (cf. Butterfield et al. 2005). To ensure sufficient spread in incidents, another 15 interviews were conducted, in total 30. This improved group-data heterogeneity regarding age, functional limitations (Table 1) and travel habits. Altogether, 469 incidents were collected: 378 negative, 85 positive and 6 both positive and negative. Ethical approval was obtained from the Stockholm Area Local Ethical Committee (2011/1169-31/5).

**Table 1:** The functional limitations (FL) of the 30 participants, classified in 3 age groups of older persons (65–91 years)

Functional limitations (FL)	1921–1929 (*n* = 6)	1930–1939 (*n* = 13)	1940–1947 (*n* = 11)	1921–1947 (*n* = 30)
Restricted mobility	5	5	7	17
Vision impairment	4	6	4	14
Cardiovascular disease	1	6	3	10
Hearing impairment	3	4	2	9
Chronic pain	1	4	3	8
Diabetes	3	2	0	5
Asthma, allergy, hypersensitivity	0	2	2	4
Attention, memory, concentration disability	2	1	1	4
Neurological disorder	1	2	1	4
Chest disease	1	0	2	3
Mental ill-health	0	1	1	2
Reading, writing or speech disability	2	0	0	2
Travel sickness	0	1	1	2
Rheumatic diseases	1	0	0	1
Sum of FL	24	34	27	85
No FL	0	1	0	1

### Data analysis

Incident categories should reflect the *causes* of the incidents (Edvardsson 1998). According to the reciprocal model of Sundling et al. (2014b), accessibility may be altered by changes in one of three variables; functional limitation/ability, barriers/facilitators, or travel behaviour. The present research focuses on identifying environmental barriers/facilitators, so the incidents are classified according to the respondents’ external^2^ attributions. If a respondent had encountered problems bringing luggage on board a train and referred to a “lack of staff” as a cause, the incident was coded as “staff”; but if reference was made to the “design of the vehicle”, the incident would be coded as “physical environment”. Some events were coded as more than one incident. For example, waiting for the bus in a “poorly lit bus station” (“physical environment”) and at the same time feeling “unsafe because of the people there” (“fellow passengers”) were coded as two separate incidents, because of the two attributions.

**Fig. 1:**
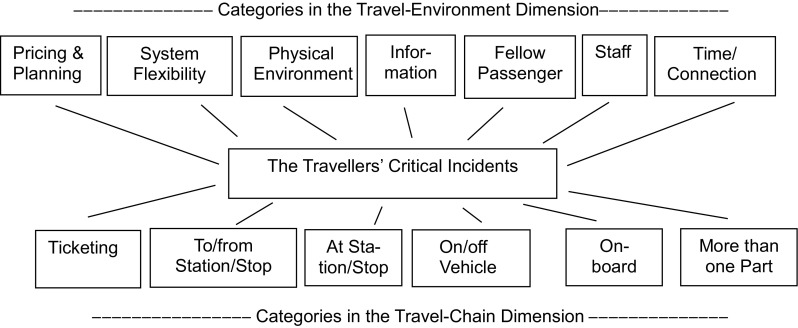
Categorisation scheme for travellers’ critical incidents, encountered due to barriers/facilitators in public transport travelling. The categories are organised in a Travel-Environment Dimension and a Travel-Chain Dimension. The critical incidents encountered are affected by functional ability and travel behaviour (Sundling et al. 2014a, b)

Two authors categorised the incidents independently. The interviewer (1st author/judge) repeatedly listened to the recorded interviews, made completing notes, and sorted the incidents according to their similarities (cf. Bitner et al. 1990) into categories organised in a “Travel-Environment” (Table 2) and a “Travel-Chain” (Table 3) Dimension (Fig. 1). Thus, each incident was sorted into one of the categories in each of the two dimensions. Each set of categories in the two dimensions was exhaustive. The dimensions and their categories were developed such that the *needs for change* in the transport system *could best be communicated* (cf. Flanagan 1954).

**Table 2:** Definitions of the set of seven categories constituting the travel-environment dimension

Categories of the travel-environment dimension	Definition/description grounded in travellers’ perception
Pricing	Price level and tariff structure that would affect the costs for travelling; e.g. tickets being perceived as expensive/inexpensive or tariff structure difficult to understand
System flexibility	How special needs or requests are met by the transport system, e.g. the possibility to book tickets without a computer; choose one’s seat; or to bring a bicycle on the vehicle
Physical environment	The physical environment in each part of the travel chain; e.g. functioning elevators and escalators in stations and vehicle design for storage of luggage
Information	The possibility and easiness to get information.
Fellow passengers	Other persons’ behaviour within the transport system (except staff). Note that this category does *not* apply to “To and from the station” in the “Travel Chain” dimension
Staff	Personal service, attitudes of staff and shortage of staff, etc. Note that this category does *not* apply to “To and from the station” in the “Travel Chain” dimension
Time & connections	Time aspects such as waiting time, punctuality and time to change to connecting travel mode

**Table 3:** Definitions of the set of six categories constituting the travel-chain dimension

Categories of the travel-chain dimension	Definition/description grounded in Travellers’ Perception
Ticketing	Incidents regarding the part of the trip concerning ticket information or purchasing
To and from station/stop	Incidents on the way to and from start or end destination station/stop areas (except for ticketing)
At station/stop	Incidents in the station/stop area (except for ticketing)
On and off vehicle	Incidents while getting on-board or off the vehicle
On-board	Incidents on-board (except for ticketing)
More than one part of the trip	Incidents that concern more than one part of the travel chain (except for ticketing)

A categorisation-coding instruction was created (1st judge). The 2nd judge then, independently, categorised the incidents identified in one interview. The two judges reached consensus through discussion. Thereafter, the 2nd judge listened to and categorised the incidents in the remaining 29 interviews. After a second discussion, the judges reached a consensus. All incidents were thus finally sorted into one of the categories for each of the two dimensions, resulting in a set of 21 combined “Travel-Environment-and-Travel-Chain” categories out of 42 possible.

The *between*-*judge reliability* was calculated using the I_r_ statistic of Perreault and Leigh (1989) that utilises the number of categories, see also Gremler (2004). In our data, the inter-judge reliability was *I*_r_ = 0.90 for the Travel-Environment Dimension and *I*_r_ = 0.88 for the Travel-Chain Dimension. Inter-judge reliabilities exceeding *I*_r_ = 0.80 are considered satisfactory. Our value of *I*_r_ ≈ 0.90 is, therefore, reassuring (Kassarjian 1977; Keaveney 1995).

## Results

The participants were 65–91 years old (AM: 76, Table 1), 24 women and 6 men. Most interviewees had a combination of functional limitations, with the number increasing with age (Table 1). Functional ability ranged from “not reduced” to “extremely reduced”. During the last 2 years, all participants had travelled by public transport, but “the kind of mode most often used” and “travel frequency” differed. Only 7 had a car in the household. Most interviewees lived in the inner part of Stockholm city or in its local suburban area. The public transport in Stockholm is relatively well developed with low-floor buses and several underground and local train lines. Various travel modes and routes were represented in the reports of the interviewees. Travels with long-distance trains and buses nationally were reported, while local travel included commuter trains, underground, local trains and buses. Trips made with a single travel mode were reported as well as connections.

Changes in travel behaviour would probably often result from more than one single incident. Some incidents are, however, more influential than others. Out of 469 collected incidents, 77 (16 %) had “highly influenced” the participants’ travel behaviour (3 on a 3-point scale); 67 of them negatively and 10 positively. We have therefore focussed on these 77 *critical* incidents; the most frequently reported were categorised in the *physical environment on board* the vehicle and *at stations/stops* followed by *pricing* and *ticketing.*

### Critical incidents in the physical environment on-board

*Lurching vehicles or under the ground environment.* Lurching vehicles induced unpleasantness and insecurity on-board buses and double-decker trains. Interviewees were afraid to fall (or, in some cases, became travel sick). Below-the-ground travelling would be unpleasant, especially if the train had stopped in a tunnel or at a station without any doors opening. Notably, only the women reported such incidents (they were in the majority). Commuter trains were appreciated because of their aboveground environment and their rapidness. If possible, some interviewees therefore travelled by commuter trains or taxi.

I avoid the fast train because of its lurch, I prefer the Inter City train. If there is no alternative, I travel by fast train (Man, aged 78).

I always choose the bus if possible, I use the underground only if it’s necessary, I feel trapped (Woman, aged 66).

*Cramped space* between seats was physically painful, e.g. in already aching knees. One interviewee had, on several occasions hurt his head badly against the luggage shelf above the seat. The spaces for luggage were perceived to be too scarce. For example, one interviewee had started using a backpack; easier to stow in the luggage compartment than a carry-on roller, but uncomfortable to carry because of balance problems. Many interviewees would have preferred to have kept their luggage close by to keep an eye on it. One participant found double-decker trains to be *“like cattle*-*trucks”,* because the carriages were cramped. The same participant would *“never get on such a train again”.*

I don’t travel 2:nd class; you never know in advance what carriage you will get. But sometimes you can get a priority seat. I always make sure I don’t pack too much (Woman, aged 71).

*Unpredictable lack of food supply* in restaurant facilities would complicate travelling. Unexpectedly, food would not be available on long-distance trains, either because there was no dining car or the food was sold out. This is a problem for persons with diabetes.

I always bring food because I don’t know if there will be any. I want to know if food can be purchased. It affects my inclination to travel (Woman, aged 67).

### Critical incidents in the physical environment at stations/stops

*Elevators/escalators out of order* is a common complaint that seriously affects the ability to travel, and the inability to get in touch with staff constitutes an additional barrier. Incidents piling up (one incident becoming the antecedent to another) may further complicate travelling. One interviewee encountered four incidents on the same trip. First, upon arrival at the station, the escalator was out of order and he, therefore, used the elevator. Second, the next escalator was also out of order. Third, the elevator was out of order, as well. He walked the stairs with great difficulties, just to discover that he had missed the train. Fourth, after reporting the errors, no one showed up during the 2 h he had spent waiting for the next train. He had experienced similar problems at several occasions. Travel behaviours had been altered in various ways for the interviewees. Some could use the stairs, although with much effort. Some could travel without a bag but not if groceries or a suitcase were to be carried. Others now allowed more time at the station in case of unexpected events. Some had ceased travelling altogether with public transport because of their experiences.

People rushed back and forth and I was stranded there. Finally, a young man helped me with my walker. But after that experience, I didn’t travel as usual, not immediately; I thought: I don’t want to be stranded again. It still affects me. I worry. Before a trip, I hardly sleep. I think of it every time. But if I must, I use the underground (Woman, aged 73).

There is no staff at the platform to help with walkers and strollers. And my back aches and I cannot lift my bag. You become more or less caged in at home (Woman, aged 80).

*Design features in station areas* are for example long escalators, perceived as vertiginous and as moving too fast. Effects on travel behaviour were e.g. to avoid travelling alone. In new designs, interviewees wished long escalators to be divided into two. One participant had fallen in an escalator. Since then, she had never travelled by underground or used escalators. Moreover, if railway platforms were cramped with people, they were perceived to be narrow and insecure. Stairs lacking bannisters were experienced as barriers. Outdoor platforms lacking shelters were too exposed to the wind and the cold.

I cannot travel alone where there are long escalators. My husband has to walk in front of me downwards and behind me upwards. If I were alone, I would take a detour and walk to another station (Woman, aged 67).

I have frequently been standing on windy, unsheltered platforms. It’s so outdated! They should build acrylic-glass shelters. I have chosen not to travel by train; I travel by bus (Woman, aged 67).

### Critical incidents in pricing and ticketing

*The cost of travelling* was perceived as either a barrier or a facilitator. This was the third most common cause for changed travel behaviour (combined category of pricing/ticketing). Travel behaviour was constrained by high prices (e.g. Wallin Andreassen 2005) or facilitated by prices perceived as low. Discounts for seniors, or unexpectedly low-prices for 1st class long-distance train tickets, both made train a possible choice or enabled frequent travelling. Common complaints were non-transparent ticket prices, fluctuating with time of booking, or better prices online than on the telephone combined with long queues at the counter. But, other travellers appreciated fluctuating prices because of the possibility of purchasing inexpensive tickets if booked well in advance or at the last minute. Some interviewees did not have a computer or found it difficult to use one; others perceived that they were “being forced” to use a computer. As an alternative to electronic solutions, they wanted personal service.

I don’t know if I am being deceived or not. If I travel by bus, I know that the price will be the same no matter when I book (Woman, aged 65).

### Other critical incidents and important events

Four categories of less frequently reported *critical incidents* were: (a) *Flexibility* in ticketing. Allowing credit card payment on the phone was viewed as a facilitator for travelling. (b) *Connections* (involving change of travel modes). Difficulties would arise especially if travelling with luggage, and/or if punctuality was low, leading to connections missed. Notably, the underground was usually considered accessible, with its frequent departures (experienced also as a security factor) and punctuality. (c) *Information* that was incorrect, unclear or contradictory could make travelling more difficult, especially if there were no staff to help out. (d) Several interviewees reported *staff*-related critical incidents caused by bus drivers. They started driving before the interviewee was seated, causing one person to fall and others being afraid to fall. Bus drivers also stopped far from the pavement, creating a high step entering or alighting the bus, especially if the kneeling function was not used. Interviewees who had experienced such incidents were the older old, especially those who used a walker. Consequences for travel behaviour was e.g. to use the Special Transport Service (a taxi service for disabled) if carrying a bag, e.g. *from* the grocery store. Travelling was facilitated if travellers knew there would be staff available to help with luggage or getting on-board. Events *prohibiting* planned trips (and therefore not categorised as incidents) were difficulties carrying luggage, climbing stairs into train, and changing trains (all of which were easier in air travel and subsequently chosen); difficulties reaching the station/stop if pavements were not cleared of snow or if the designated accompanier from the municipality did not show up. One respondent fell outside our inclusion criteria because of such problems, during the last 2 years, she had been unable to travel *by railway*. Some interviewees avoided buses but travelled by underground. For others, it was the other way around, mainly because of the dependence on elevator or escalator for reaching the underground.

They don’t kneel the bus. Will I be able to travel by bus if I carry bags? I cannot jump with my walker. They say there is a ramp but I have never seen it being used, do you think the driver would use it?! Sometimes you see wheelchairs—I think they are brave. Those were not older people, they were the young. I shouldn’t have to use the Special Transport Service; you should be able to take the bus, even if you are disabled (Woman, aged 76).

## Discussion

Older people experience critical incidents in all parts of the travel chain, so the entire journey is important. The study reveals that critical incidents are most often encountered in the physical environment on-board vehicles and at stations/stops. The categorisation system that emerged from our empirical data was useful for structuring our results and in deepening our understanding of the experiences of older adults in public transport.

Sundling’s et al. (2014b) conceptual accessibility model was used to study links between barriers/facilitators (perceived as critical incidents) and subsequent travel behaviour. In the model, functional ability and travel behaviour may be altered by changes in barriers/facilitators. Our findings highlight the importance of staff for service and security in different parts of the trip. This is consistent with previous research (Hine and Scott 2000; Green et al. 2014), showing that staff may help bridging the gap between the traveller’s functional ability and environmental barriers (Rosenkvist et al. 2010).

Employee behaviour is important for satisfaction with public transport (Friman et al. 2001; Friman and Gärling 2001). Bus-driver behaviour was a principal reason for difficulties when getting on/off buses—a barrier identified also in earlier studies (Hine and Scott 2000). Wretstrand et al. (2009) showed that even if low-floor buses were in use, alighting might still be difficult for older people. Our study shows that functional ability is lowered when the kneeling function is not used and that irregular use complicates older people’s travel decisions.

This study confirms that ticket prices constitute barriers for travelling. Earlier studies show that, for older adults and those with lower income, ticket prices affect travel behaviour (Hine and Scott 2000) and lower ticket prices improve customer perceptions of quality in public transport (Eboli and Mazzulla 2010). For older people, travel costs are often more important than e.g. travel time (Su and Bell 2009; Su et al. 2009), while delays are major barriers for younger persons (Friman et al. 2001; Friman and Gärling 2001; Beirão and Sarsfield Cabral 2007). Our results support this relation. A retiree may have a more strained private economy than earlier in life, but more time to spend. Some interviewees concluded that they have fewer appointments to keep so that delays are now not as serious as when they were at work. Complex journeys often referred to physical complexity rather than cognitive (cf. Rosenkvist et al. 2009), possibly because restricted mobility was the most common functional limitation in our sample. Neither frequency of service, often found important for younger travellers (Hine and Scott 2000; Eboli and Mazzulla 2008) nor distance to bus stop, for older travellers (Schmöcker et al. 2008), were found to be essential for travel behaviour. Most of our interviewees were living in high-density areas, with short distances to public transport. Thus, distance was not restricting their functional ability.

Asking about past events always means exposure to memory biases (Kahneman 2000). This is especially true for older people and is a disadvantage of all retrospective self-reports that would rely on memory. However, Friman and Gärling (2001) showed that satisfaction with public transport did not differ with how recent the critical incident was. The CIT has been the most commonly used method to explain changed customer behaviour (Edvardsson and Roos 2001). The interviewees live in different municipalities and differ as regards age, gender, functional limitation/ability, and travel modes used. Therefore, before generalising our results, our recommendations should be interpreted cautiously. On the other hand, the choice of interviewees and consequential heterogeneity should work against effects of serious selection-bias.

### Policy and practice

Our key findings show that

- If provided along the travel chain, more *personal service* would facilitate travelling.
- *At stations*, swift maintenance and repair of elevators and escalators would help improve predictability, as would surveillance and telephones for assistance in unexpected situations.
- *Bus drivers* should stop near the pavement; use the kneeling function; wait until older travellers are seated; drive evenly; and avoid lurching, sudden stops/accelerations.
- *Luggage handling* would be facilitated with check-in possibilities at railway stations. Future carriages should ideally be designed for travellers’ seat-visible storage of own luggage. Travellers need to be “on guard” because of the (perceived) risk of theft. The possibility to relax has been found to be a reason for choosing public transport instead of the car (Hine and Scott 2000).
- Clear and accurate *information* is necessary, such as audible and visual announcements of travel-mode changes. Important signs and displays should be rapidly updated, replaced, or improved.
- *Ticketing* should be flexible, e.g. providing comparable alternatives to web-based purchases. Coordination among operators, regarding timetables and ticketing would facilitate ticketing (cf. Hine and Scott 2000).
- *Different kinds of end users* should be involved in *planning and design* to elucidate their various needs.

## Footnotes

1 A taxi service for persons with functional limitations.

2 Cause of incident placed outside the person.
